# An Atypical Presentation of Epstein-Barr Virus Associated Infectious Mononucleosis Mistaken for Pyelonephritis

**DOI:** 10.7759/cureus.7583

**Published:** 2020-04-07

**Authors:** Cassandra Kien, Kavitha Ganta

**Affiliations:** 1 Biological Sciences, Arizona State University, Tempe, USA; 2 Nephrology, Raymond G. Murphy Veterans Affairs Medical Center, Albuquerque, USA

**Keywords:** epstein-barr virus infection, infectious mononucleosis, abnormal urinalysis, lymphadenopathy, fever, splenomegaly, urticaria, pharyngitis, acute interstitial nephritis, neck mass

## Abstract

Infectious mononucleosis, a syndrome characterized by the triad of pharyngitis, fever, and lymphadenopathy, is caused in the majority of cases by Epstein-Barr virus and usually presents in adolescents and young adults. The disease is for the most part self-limited with full recovery; however, life-threatening complications can occur. Manifestations of Epstein-Barr virus associated infectious mononucleosis can be variable and at times atypical, leading to a delay in diagnosis and consequently unnecessary tests and treatment. We present a case of infectious mononucleosis from Epstein-Barr virus in a female college student who was admitted to the hospital with the initial diagnosis of pyelonephritis. This diagnosis was made based on an abnormal urinalysis, including the presence of white blood cells, red blood cells, and protein, in the setting of high fevers, cough, abdominal pain, left costovertebral tenderness, and an unexplained left neck mass. A monospot was negative two days prior. Renal involvement in Epstein-Barr virus infection is not common and bridges the spectrum from asymptomatic urinary abnormalities to acute renal failure, with acute interstitial nephritis being the most frequent pathological finding. Our patient received corticosteroids and albuterol for a worsening cough, in addition to supportive care. Despite steroid therapy, she developed a debilitating, protracted urticarial rash, also thought to be caused by the Epstein-Barr virus infection. Our case highlights the varied and complex constellation of findings sometimes seen in Epstein-Barr virus infectious mononucleosis. Like in our patient, pharyngitis, a part of the hallmark triad of symptoms characterizing infectious mononucleosis, is not always present, and the monospot may be negative. A high degree of suspicion, as well as recognition that multiple organ systems may be involved in Epstein-Barr virus associated infectious mononucleosis, is required to make the proper diagnosis.

## Introduction

Epstein-Barr virus (EBV) is one of the most common viral infections worldwide and is the principal virus responsible for the clinical syndrome known as infectious mononucleosis (IM) [1]. EBV, a double-stranded DNA virus, is a member of the herpesvirus family. Studies have shown that EBV infection experienced during early childhood is mostly asymptomatic, whereas a majority of adolescents and young adults with initial exposure to EBV experience IM [2]. More than 90% of adults have serological evidence of EBV. However, the epidemiology of EBV infection may be changing. Children are acquiring EBV infection later in age in industrialized countries [2-5]. This is important given the recognized association between the age of primary EBV infection and disease severity. This complex relationship between the age at which infection occurs and acute illness is not fully understood, although the changing responsiveness of the immune system with age is evoked as an important factor [3].

EBV is transmitted mainly through oral secretions [6]. Symptoms of infection typically occur between 30 and 50 days after exposure [4,6]. Once EBV infection occurs in an immunocompetent individual, regardless of presentation, it usually remains in a quiescent state. Nevertheless, it may reactivate occasionally. This reactivation in most cases does not cause illness, yet shedding of EBV after reactivation may transmit the virus to EBV naive individuals, accounting for why some patients with EBV-associated IM cannot identify an exposure to the virus [7,8].

IM, historically known as glandular fever, typically presents with the triad of fever, lymphadenopathy, and pharyngitis accompanied by malaise and fatigue. An increase in lymphocytes, some of which may be atypical, is found in the peripheral blood. Mild elevations of aminotransferases are common as well in IM [1-6,9]. EBV-related IM should be in the differential diagnosis of any teen or young adult presenting with a fever (usually low-to-moderate grade), sore throat, and lymph node enlargement, which are all archetypical findings in EBV-induced IM. The diagnosis is frequently made when heterophile antibodies are identified in a patient presenting with symptoms and findings consistent with IM [3,10]. Heterophile antibodies are immunoglobulin M (IgM) antibodies produced by the polyclonal expansion of B cells infected by EBV. These antibodies, however, are not directed against EBV but are rather directed against antigens found on erythrocytes from unrelated animals such as sheep, horse, and cow [10]. EBV-specific antibodies are also produced along with these heterophile antibodies and are used to confirm the diagnosis of an EBV infection in the setting of a negative result for heterophile antibodies [10]. The most recognized heterophile antibody test used to rapidly diagnose EBV disease is the “monospot” [11]. This latex agglutination assay detects antibodies directed against horse erythrocytes and is thought to be highly specific and sensitive for EBV infection in adolescents and adults, although in the first week of infection up to 25% of patients with EBV IM will have a negative monospot [12]. Additionally, young children may not develop heterophile antibodies [13]. When the monospot is positive in the right clinical setting, the diagnosis is confirmed without the need for specific EBV antibody testing. The course is usually uneventful with most symptoms, except fatigue, resolving within weeks, and long-lasting immunity achieved.

IM caused by EBV infection, however, can sometimes present with protean findings. Any organ system is at risk of being infected with EBV. Abnormalities including hemophagocytic lymphohistiocytosis, splenomegaly, hepatitis, pancreatitis, encephalitis, myositis, myocarditis, and cholecystitis, as well as skin, lung, and kidney involvement have been reported with IM from EBV [1,3,12]. Malignancy may also occur as a long-lasting after-effect of EBV infection in certain individuals, and recent research supports an association between EBV infection and multiple sclerosis [14]. Despite the burden of EBV infection, a vaccine against EBV is not yet available [2,14]. When IM acquired from EBV infection presents with an atypical constellation of findings, as seen in about 15% of young adults, the correct diagnosis may be difficult to make, and sometimes IM is confused with other illnesses [1,12]. Although almost all individuals with IM recover with only supportive care, misdiagnosis may result in unnecessary testing and treatment, along with a lack of identification of rare but potentially serious complications of EBV-associated IM [1,3,12]. We present a case of IM from EBV infection with atypical findings that was initially misdiagnosed as pyelonephritis.

## Case presentation

A 20-year-old female college student in Arizona presented to the emergency department with new-onset abdominal pain along with five days of a non-painful left neck mass, a dry cough, and shaking chills with fevers to 40 degrees centigrade despite taking ibuprofen alternating with acetaminophen. Her history was significant for hypothyroidism from Hashimoto’s thyroiditis and von Willebrand disease, both diagnosed eight years prior. She was taking levothyroxine, and used aminocaproic acid as indicated. Birth control was achieved through a long-standing hormonal intrauterine device, and consequently she was not menstruating. Rarely she would experience bronchospasm with upper respiratory infections and would briefly use an albuterol inhaler when needed. Social history, family history, and travel history were unremarkable. She denied using tobacco or street drugs. Our patient worked part-time as a medical scribe, carried a full college class load, and volunteered as a basic science research assistant on campus.

**Figure 1:**
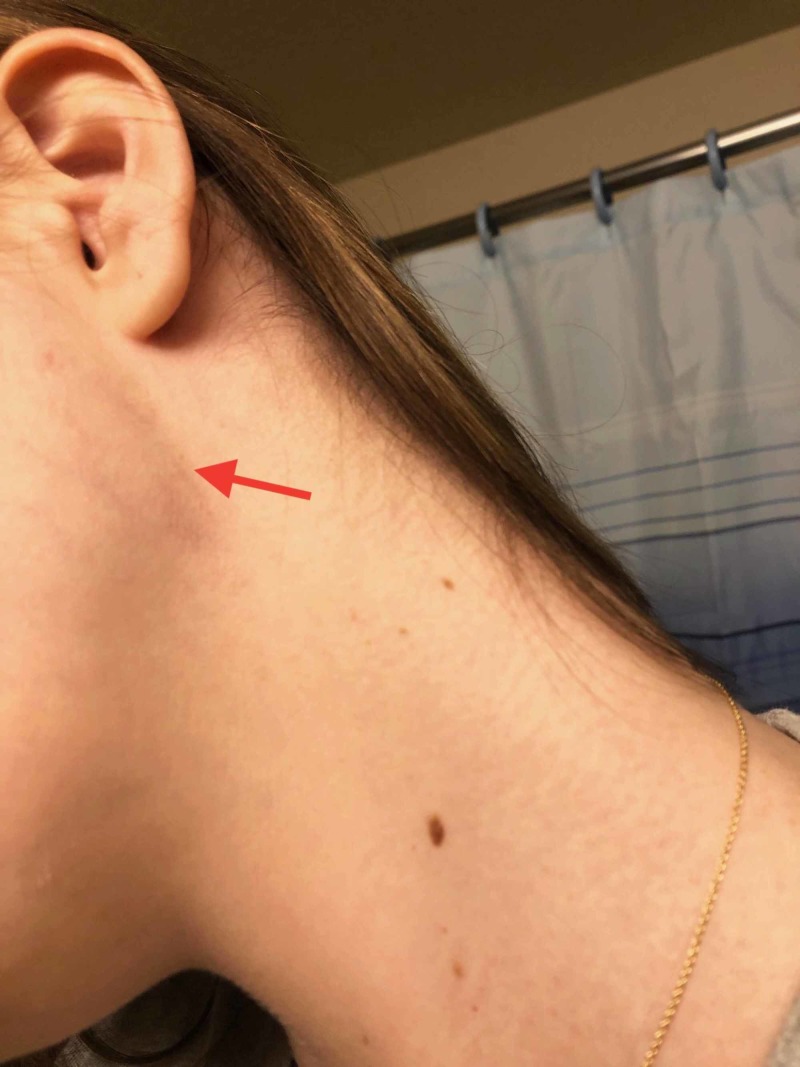
Left neck mass diagnosed as anterior cervical lymphadenopathy

She was seen two days earlier at an urgent care facility for generalized malaise, intermittent fevers with chills, a non-productive cough, and a left neck mass. At that time, she denied a sore throat, abdominal pain, nausea, vomiting, diarrhea, dysuria, headache, neck stiffness, travel, or exposure to others who were ill. Her temperature was 38.9 degrees centigrade, and she appeared moderately ill. A non-tender left infra-auricular neck mass was present, which was thought to perhaps be a lymph node (Figure 1). Examination of her throat showed normal-appearing tonsils. There was no pharyngeal erythema, petechiae, or exudate. Her lungs and abdomen were unremarkable. Monospot and rapid strep screen were negative. She was diagnosed with a non-specific viral illness. Because of the possibility of an atypical bacterial infection, she was started on azithromycin.

Her symptoms waxed and waned. However, she developed abdominal pain with continued fevers. This prompted her to seek further evaluation in the hospital emergency department. For a second time, she denied having a sore throat or dysuria. Her physical examination revealed an ill-appearing young woman with a temperature of 38.9 degrees centigrade, heart rate of 109 beats per minute, blood pressure of 116/75 mm Hg, and oxygen saturation of 94% on room air. Non-tender right anterior cervical lymph nodes were noted. A non-tender left neck mass without discrete lymphadenopathy was also described. Her pharynx and lung examination were once more normal. Bilateral upper abdominal tenderness with normal bowel sounds was noted, as was left costovertebral tenderness. No skin rash was present. White blood count was 7,300 per mL with 41% neutrophils, 45% lymphocytes, and 10% monocytes. Hematocrit was 42%, and platelet count was 102,000 per μL. No atypical lymphocytes were reported. Serum chemistries, including creatinine and liver function tests, were normal. Urinalysis was significant for 11-20 red blood cells and 6-10 white blood cells per high power field. Leukocyte esterase and nitrite were negative. Urine protein was > 500 mg/dL, and specific gravity was 1.028. Further quantification of the urine protein concentration was not performed. Blood cultures were drawn, and the patient was admitted to the hospital with the diagnosis of pyelonephritis with possible bacteremia. She received intravenous hydration and ceftriaxone.

**Figure 2:**
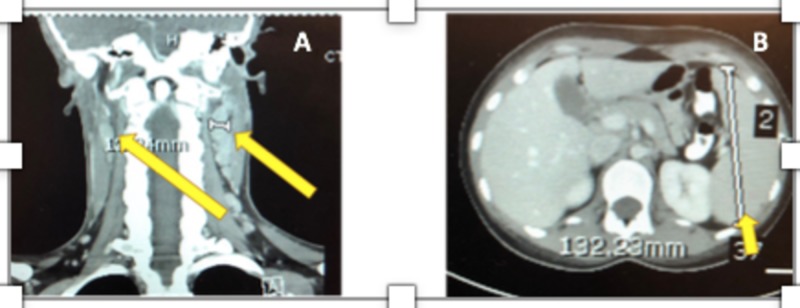
Computed. Enlarged cervical lymph nodes (A). Enlarged spleen (B).

During the first day of hospitalization, computed tomography (CT) of the neck, lungs, abdomen, and pelvis revealed bilateral cervical and supraclavicular adenopathy, and an enlarged spleen measuring 13 centimeters (Figure 2). Chest radiograph and renal ultrasound were normal. Her temperature reached 40.2 degrees centigrade, and systolic blood pressure decreased to 94 mm Hg. She continued to experience a persistent non-productive cough and increasing upper abdominal pain requiring intravenous morphine. Multiple serologies, including respiratory viruses, EBV, human immunodeficiency virus, hepatitis C, and coccidioidomycosis, were obtained. On the second hospital day, fevers, although not as high, cough, and abdominal pain continued. Blood and urine cultures were negative, and another chest radiograph taken was normal. Antibiotics were stopped. Repeat complete blood count revealed a hematocrit of 31% and a platelet count of 84,000 per μL. Total bilirubin was 1.3 mg/dL, international normalized ratio (INR) was 1.2, and lactate dehydrogenase 328 IU, all only minimally elevated, as was ferritin at 318 ng/mL. D-dimer was normal, and hemolysis was not thought to be likely. Epstein-Barr viral capsid IgM index was 3.8, with an index of <0.08 being negative, whereas Epstein-Barr viral capsid immunoglobulin G (IgG) was not detected. These indicated an acute EBV infection [3]. Coccidioides complement-fixing (CF) antibodies detected by enzyme immunoassay (EIA) were reported as indeterminate, whereas tube precipitin (TP) antibodies by EIA were identified and reported as reactive. CF and TP antibodies measured by EIA are screening tests for coccidioidomycosis and measure mainly IgG and IgM antibodies, respectively. False-positive results occur when testing for Coccidioides TP antibodies using EIA [15]. The diagnoses of IM from primary EBV infection and possible coccidioidomycosis, also known as valley fever and endemic to the area where the student lived, were made [3,15]. Antivirals were considered but not administered, and oral fluconazole was started awaiting confirmatory serologies for coccidioidomycosis.

During the third day of hospitalization, her cough persisted and was treated with and responded to nebulized albuterol and solumedrol. She was discharged after four days in the hospital, afebrile, with improving complete blood count, complaining only of fatigue. Her cough and abdominal pain had resolved. Discharge instructions included rest, a prednisone taper, and fluconazole. Confirmatory Coccidioides IgG and IgM antibodies by immunodiffusion were both reported as negative one day after discharge, and fluconazole was stopped.

**Figure 3:**
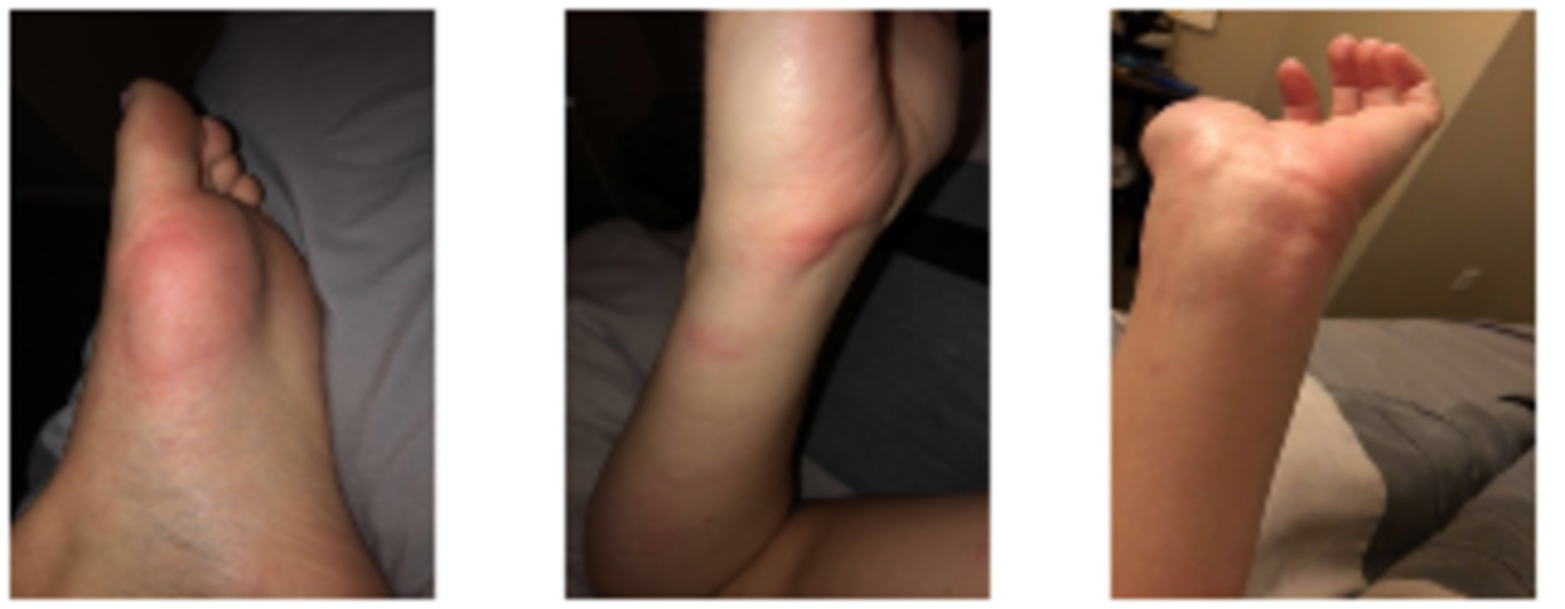
Urticarial rash while taking prednisone.

A day and a half after discharge, our patient developed debilitating urticaria despite taking prednisone (Figure 3). Testing for cold urticaria was negative. Initial management with diphenhydramine and topical steroid while continuing the seven-day prednisone taper did not alleviate symptoms. The urticarial rash was ultimately controlled and resolved with fexofenadine after two weeks. Three weeks after initial symptoms, the patient had a normal urinalysis, complete blood count, bilirubin, and INR. Alanine aminotransferase was 47 IU/L, with the normal upper limit being 46 IU/L. Fatigue persisted for eight months.

## Discussion

IM, most commonly occurring from EBV, is usually a self-limiting illness that presents with the recognized constellation of findings: fever, pharyngitis, and adenopathy [1,14]. Complications involving almost any organ system may occur and, as in our case, confound the diagnosis [1,3,12,16,17]. Our patient sought medical care early in her course when IM was considered. A monospot was performed, with the result being negative. This may have been a consequence of our patient presenting early in the course of her infection. The early stage of disease was later borne out by her EBV specific serologies, with anti-VCA IgM being highly positive and Anti-VCA IgG being negative [3]. Additionally, our patient exhibited antibodies suggestive of coccidioidomycosis infection by screening serology. We can hypothesize that the polyclonal B-cell amplification resulting from EBV infection produced antibodies detected by the EIA serologic screening tests used for diagnosing coccidioidomycosis.

When she presented to the emergency department with fevers, higher than those usually occurring with IM, an abnormal urinalysis, and abdominal as well as left-sided costovertebral angle tenderness, she was diagnosed with pyelonephritis. Pyelonephritis, however, did not explain all her findings, and the diagnosis of EBV-related IM was made in consultation with infectious disease specialists. Urine abnormalities were attributed to EBV IM, although proteinuria was not quantitated and most likely represented sub-clinical acute interstitial nephritis and not bacterial pyelonephritis.

Our case highlights some common as well as uncommon findings in IM acquired from EBV. Our patient presented without pharyngitis, without atypical lymphocytosis, and without elevated amino aminotransferase, as observed in most patients with IM from EBV. However, she did complain of fevers and a neck mass, as well as cough and abdominal pain, which are sometimes observed in IM. She developed a significant worsening of thrombocytopenia and a new anemia, which, in part, may have been due to hydration. However, these laboratory abnormalities, when minor, are also not uncommon with EBV-associated IM [1]. Dunmire et al., in a study of college students, reported that 98% of students with primary EBV infection and IM had a sore throat. Enlarged or tender cervical lymph nodes were found in 88%, whereas a febrile feeling was described by only 53% of students [2]. This may be because fever in IM especially early on is usually low-to-moderate grade and may not be accompanied by shaking chills, one of the hallmarks of a significant febrile occurrence. Cough or other upper respiratory symptoms were described in 48% of the students studied. Abdominal pain was not as common a complaint, occurring in only 15% of college students in the report by Dunmire et al. [2]. Abdominal pain in EBV-associated IM is especially worrisome in that it may herald splenic rupture, a rare but life-threatening complication of IM [1,2]. Other serious complications include airway obstruction, liver failure, and renal failure, as well as the even rarer immunologic and central nervous system involvement [1,2,16,17].

Kidney involvement in IM occurs infrequently. Acute renal failure is extremely rare, but when it occurs it may require renal replacement therapy [16,17]. When acute renal insufficiency occurs, by far the most common finding on renal biopsy is acute interstitial nephritis. A few cases of glomerulonephritis and other diseases of the kidney have also been described in the literature [16]. Urinary abnormalities in the setting of IM were first reported in 1946, where 3% of 556 military recruits diagnosed with IM had microscopic hematuria and proteinuria [18]. Another study by Lee and Kjellstrand published in 1978 reported an 11% and 14% incidence of hematuria and proteinuria, respectively [19]. The reporting of kidney involvement in IM may be underestimated given that the vast majority of urinary abnormalities identified, as in our case, are of little clinical consequence. The findings of sterile pyuria, microscopic hematuria, and proteinuria in the setting of an acute illness suggest an interstitial inflammatory process, though in the context of normal renal function, renal biopsies would rarely, if ever, be performed. The spectrum of abnormalities caused by renal interstitial inflammation in IM ranges from abnormal findings in urinalysis to acute kidney injury requiring hemodialysis. It is important to recognize the variety of presentations indicating kidney involvement in IM. Acute renal failure and acute interstitial nephritis with significant renal insufficiency are usually easily identified as complications of EBV-related IM. However, asymptomatic urinary abnormalities may lead to a misdiagnosis of urinary tract infection, as in our patient. This is especially true when, as in our case of IM, presentation is atypical. The findings in our patient of high fever, abdominal pain, and left costovertebral angle tenderness after correct diagnosis were presumed to be from EBV infection with splenic involvement along with the possible role of mesenteric lymphadenitis or liver and gallbladder involvement [2]. They were not due to pyelonephritis.

Our patient’s condition worsened in the first 24 hours of hospitalization during which she experienced continued high fevers, hypotension, persistent cough, and worsening abdominal pain. At that point, the antiviral agent acyclovir was considered but not administered given that studies have shown that administration of this category of drug, despite reducing viral shedding, had no effect on the degree or duration of clinical symptoms [12]. Corticosteroids were administered to our patient along with the bronchodilator albuterol for her persistent cough that was thought to be part of her IM syndrome. Corticosteroid use for IM in the setting of EBV is debatable. These agents are not recommended for the typical case of IM. However, they may decrease lymphoid and mucosa edema, resulting in some improvement, and are strongly recommended in the setting of airway obstruction [14]. Our patient improved significantly after receiving methylprednisolone; however, its role in her recovery is not clear.

Upon discharge, as her oral corticosteroid was being tapered, an acute debilitating urticarial rash erupted. About 10% of patients with EBV-induced IM have a skin rash [20]. A maculopapular, usually non-pruritic exanthem, thought to be directly related to the virus, involves the torso and upper arms first followed by the rash appearing on the face and forearms a few days later. This rash is different from the well-known pruritic morbilliform rash occurring with the use of beta-lactam antibiotics and other antibiotics in the setting of EBV-related IM [20]. Less frequently, petechial rashes and acute urticaria, as in our patient, including cold urticaria have been described as occurring with herpes virus infections including EBV [20]. Our patient’s urticaria appeared 12 days after initial symptoms while on a prednisone taper, four days after receiving ceftriaxone for 48 hours, and resolved two weeks after occurring. The remainder of her course was as expected for most cases of EBV-associated IM with full recovery, except for persistent but improving fatigue.

## Conclusions

Our case underscores the importance of recognizing that IM caused by EBV may present without the trademark pharyngitis. Additionally, it points out the drawbacks of using the monospot to diagnosis IM early in the disease presentation. Furthermore, our case emphasizes that the features of EBV-related IM may include findings that are uncommon and perhaps not as widely recognized as being associated with the infection, leading to misdiagnosis and, in certain cases, life-threatening complications. Our patient recovered with supportive care, which is the mainstay of treatment for IM. Receiving parenteral corticosteroids may have influenced her course; however, their role in our patient is unclear. EBV infection is pervasive across the globe. It is best known for causing IM in teens and young adults. And as our case illustrates, the diagnosis of EBV disease may be evasive, requiring a high degree of suspicion.
